# Percutaneous Radiofrequency Ablation Versus Robotic-Assisted Partial Nephrectomy for the Treatment of Small Renal Cell Carcinoma

**DOI:** 10.1007/s00270-016-1417-z

**Published:** 2016-07-19

**Authors:** Maria Pantelidou, Ben Challacombe, Andrew McGrath, Matthew Brown, Shahzad Ilyas, Konstantinos Katsanos, Andreas Adam

**Affiliations:** 1 Department of Interventional Radiology, Guy’s and St. Thomas’ Hospitals, NHS Foundation Trust, King’s Health Partners, London, SE1 7EH UK; 2 Department of Urology, Guy’s and St. Thomas’ Hospitals, NHS Foundation Trust, King’s Health Partners, London, SE1 7EH UK

**Keywords:** Radiofrequency ablation, Robotic partial nephrectomy, Renal cell carcinoma, Local recurrence, Disease-free survival

## Abstract

**Introduction:**

The authors compared the oncologic outcomes of radiofrequency ablation (RFA) with robotic-assisted partial nephrectomy (RPN) for the treatment of T1 stage renal cell carcinoma (RCC).

**Materials and methods:**

This was a retrospective data analysis of a high-volume single tertiary centre. Patients were treated with RFA or RPN following multidisciplinary decision making. Only histologically proven RCCs were included. Baseline demographics were collected, and PADUA scores of tumour features were calculated to standardize baseline anatomy. Peri-operative complications, kidney function and oncological outcomes were compared.

**Results:**

Sixty-three cases were included in each group. Baseline renal function was poorer in RFA, and 16/63 RFA patients had tumours in single kidneys compared to 1/63 RPN cases (*p* < 0.001). Length of stay was shorter in RFA (1 vs. 3 days, *p* < 0.0001). Post-procedure renal function decline at 30 days was significantly less in RFA [(−0.8) ± 9.6 vs. (−16.1) ± 19.5 mls/min/1.73 m^2^; *p* < 0.0001]. More minor complications were recorded in RPN (10/63 vs. 4/63, *p* = 0.15), but local recurrence was numerically higher in RFA (6/63 vs. 1/63, *p* = 0.11). Disease-free survival (DFS) was not significantly different (adjusted HR = 0.6, 95 % Cl 0.1–3.7; *p* = 0.60). Increasing tumour size was an independent predictor of local recurrence (adjusted HR = 1.7; 95 % Cl 1.1–2.6 per cm; *p* = 0.02).

**Conclusions:**

Both RPN and RFA offer very good oncological outcomes for the treatment of T1 RCC with low peri-operative morbidity and similar oncologic outcomes. RFA demonstrated fewer peri-operative complications and better preservation of renal function, whereas RPN had an insignificantly lower local recurrence rate. RFA should be offered alongside RPN for selected cases.

## Introduction

With increasing numbers of renal cell carcinomas (RCC) being diagnosed annually, clinicians and researchers are continuously looking for ways to evaluate and find the most optimal treatments for RCC [1]. Current clinical practice has moved a long way in the past decade from open nephrectomies to laparoscopic partial nephrectomies and a current focus on robotic-assisted partial nephrectomies and other nephron sparing procedures such as radiofrequency thermal ablation [2]. This trend has evolved due to an effort to reduce peri-operative complications and maximise residual kidney function [3]. Current clinical practice dictates that partial nephrectomy remains the treatment of first choice for T1 small renal tumours (<7 cm) and that radiofrequency ablation should be reserved for a patient population deemed unfit for surgery, such as elderly patients, those with significant comorbidities, those with positive family history of recurrent tumour growth and those with solitary kidneys [4, 5].

Despite the several promising advantages offered by thermal ablation and other minimally invasive nephron- sparing techniques, clinicians hesitate to offer it as first-line treatment due to the uncertainty relating to its long-term oncological outcomes [1, 4]. This may be partly due to the fact that there is a lack of long-term follow-up and comparison of oncological control in patients undergoing radiofrequency ablation versus those undergoing partial nephrectomy [6]. A preliminary search of the literature has revealed only a small number of studies that examined long-term outcomes and directly compared RFA with RPN for the treatment of RCC [1–3, 6, 7]. There is a significant need to compare the mid- to long-term oncological and functional outcomes of RPN versus RFA in an effort to establish the efficacy of the two treatments. Such a comparison will make it possible to assess whether the current practice is optimal and to determine whether a less invasive approach should be pursued.

## Materials and Methods

### Patient Sample

**Fig. 1:**
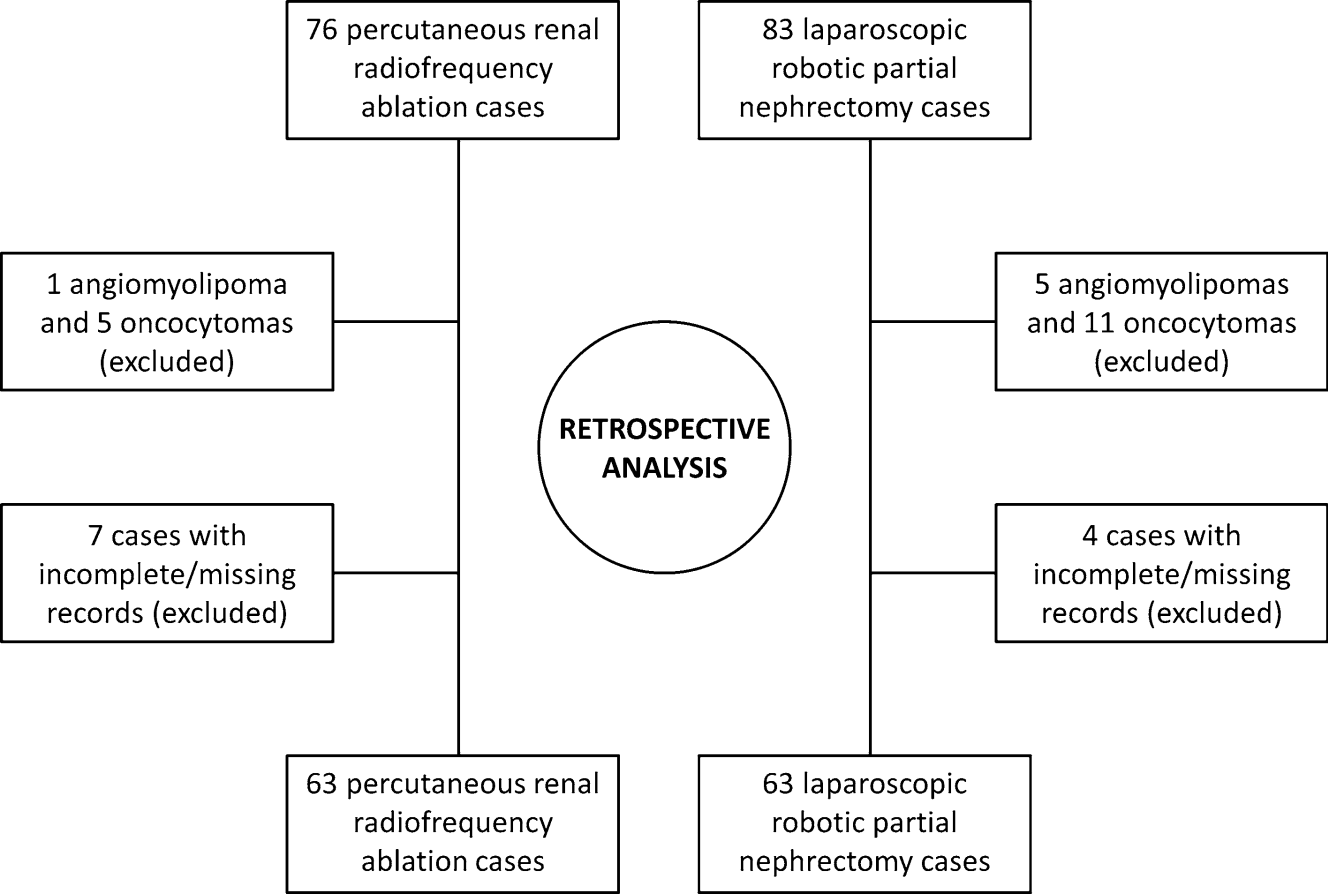
Flowchart of retrospective analysis of RFA and RPN cases from the RCC database maintained in a tertiary teaching hospital centre

This was a retrospective data analysis (clinical audit) of the RCC databases of a single tertiary centre and was approved by the Institutional Review Board for quality improvement purposes. Patients were triaged to either RFA or RPN according to the decision of the multidisciplinary meeting on the basis of tumour size, underlying comorbidities and patient preference. In our institution, patients with stage 1a renal tumours (<4 cm) are routinely offered either RFA or RPN and consented accordingly; patients with single kidneys and/or underlying comorbidities are most often offered RFA, whereas patients with larger tumours (>4 cm; stage 1b) are routinely treated with RPN or open partial nephrectomy or laparoscopic radical nephrectomy, with thermal ablation reserved for frail patients or those with underlying comorbidities. Large renal tumours treated with open partial or radical nephrectomy were not included in the present audit. In addition, only histologically proven RCCs were included in our analysis (Table 1). Benign tumours, such as angiomyolipomas and oncocytomas were excluded from this study. Data cleaning was subsequently performed to remove any duplications, outliers and cases lost to follow-up. In total, 159 cases were audited; 22 cases with benign tumour histology (angiomyolipomas and oncocytomas) and 11 cases with incomplete and/or missing records were excluded from further analysis. Finally, 126 cases were identified to fulfil the inclusion criteria (Table 1), sixty-three of whom underwent robotic-assisted partial nephrectomy (RPN) and sixty-three of whom underwent radiofrequency ablation for T1 RCC between the 5 December 2005 and 23 November 2013 (study flowchart; Fig. 1). Patient comorbidities were evaluated using the ASA (American Society of Anaesthesiologists) complications score [8]. The presence of solitary kidney in the two groups and the level of baseline renal function was also analyzed.

**Table 1:** Inclusion and exclusion criteria for cases selection

Inclusion criteria	Exclusion criteria
Histologically confirmed RCC	Benign renal cysts All types of benign tumours (e.g. AMLs)
T1 stage RCC (<7 cm)	>7 cm renal tumours
Available follow-up	Lost to follow-up cases
Available baseline pre-procedure CT imaging	No post-procedure follow-up imaging
Robotic partial nephrectomy (RPN)	Other nephrectomy techniques (e.g. open or laparoscopic)
Percutaneous radiofrequency ablation (RFA)	Other types of ablation (e.g. cryoablation)

### Treatment Techniques

RPN was performed laparoscopically using the da Vinci® Robot, Si Dual console as described elsewhere [9, 10]. Briefly, RPN starts by laparoscopically placing the robotic ports and camera in the patient’s abdomen and reflecting the bowel away from the surgical site. The renal vessels are then recognized and protected using slings. Following this, the kidney is mobilized by the surgeon, and the renal fat is reflected completely, except for over the tumour margins. Subsequently, the renal vessels are clamped when recording of warm ischaemic time (WIT) begins. The tumour is then resected and a deep tumour biopsy is sometimes performed. Renorrhaphy then follows, aiming to minimize the WIT to less than 20 min [11]. This group represented the initial series of RPN at our institution.

Radiofrequency ablation was performed percutaneously under CT guidance using Covidien cool-tip electrodes, which can produce a frequency of 480 Hz and power output of 200 W as described elsewhere [12, 13]. Straight monopolar electrodes were used, with a 17G needle. These electrodes have an internal cooling system, which allows slower rate of heating of nearby tissue and avoids carbonisation. A 17G needle was inserted under CT guidance and ablation times aimed to produce an ablation zone expanding 0.5–1.0 cm beyond the CT-determined tumour diameter [12]. All tumours were ablated for at least one cycle of up to 12 min in duration per instructions for use. Transcatheter embolization is not routinely offered in our institution as combination therapy for large-stage Ib renal masses. Instead, multiple overlapping cycles are applied in cases of tumours larger than 3 cm in order to produce an additive ablation volume of the required size [12]. Prior to each ablation, a percutaneous renal biopsy was performed using an 18G TruCut needle for histological analysis [5]. We do not usually ablate the percutaneous tract because we do not consider the risk of tract seeding to be high and are unconvinced about the effectiveness of this approach. However, in all cases, there is overlap of the ablation volume with some of the surrounding perirenal fat in order to obtain a healthy tissue margin.

After RFA, contrast-enhanced CT was performed the next day to assess the technical result of the ablation procedure. Follow-up included contrast-enhanced computed tomography in patients who had RFA or transabdominal ultrasound in those treated with RPN every 4–6 months during the 2 years and annually thereafter. In cases of local recurrence, the radiological diagnosis was based on recurrent contrast enhancement, and repeat ablation or open/laparoscopic nephrectomy was performed without repeat biopsy [5].

### Baseline Variables

The PADUA (pre-operative aspects and dimensions used for an anatomical) classification was used to score the renal tumours treated by both techniques [14, 15]. It was decided that calculating a PADUA score for both RFA and RPN groups would offer standardization of baseline anatomy among the two groups and allow direct comparison. Under the PADUA classification, renal tumours were divided and scored according to six anatomical characteristics: tumour’s longitudinal classification, the location of its margin, its relationship with the renal sinus and the collecting system, and the parenchymal depth and size [14, 15]. Brilliance™ Workspace Portal software V.2.6.1.5 (Philips), CT Workstation, was used to reformat the renal CT images and obtain the sagittal and multiplanar views necessary for the calculation of the PADUA score. The PADUA classification did not only enable us to standardize and compare the RCC tumours between the two groups but also allowed a reliable prediction of peri-operative risks. Higher PADUA scores are associated with greater peri-operative risks [16]. For example, a tumour with a PADUA score of 12a, which is entirely endophytic and invading the urinary collective system will be more challenging to excise or ablate than a tumour with a score of 7a, which is >50 % exophytic and is remote from the urinary collecting system [15].

Peri-procedural variables were retrospectively analysed for both RFA and RPN cases in order to establish whether there were any significant differences in the short- and mid- to long-term post-operative complications using the Clavien complications grading system [17]. Other parameters investigated included serum biochemistry, baseline and post-operative renal function and differences in haemoglobin plasma concentration pre- and post-procedure. Serum creatinine was measured within 30 days post-procedure. Post-intervention follow-up consisted of clinical history taking, physical examination, establishing the existence of any short- or mid- to long-term complications post-operatively and regular contrast-enhanced CT examinations. The following parameters were analysed at follow-up: signs of residual disease or local recurrence (including tract seeding during RFA), new tumour growth (in the same or other kidney), metastatic disease and whether the patient received any other treatment. The primary purpose of this study was to compare the disease-free survival (DFS) between the two groups.

### Endpoints and Statistical Methods

Clinical endpoints were used in conjunction with definitions applied by the National Cancer institute (NCI) and the European Association of Urology (EAU) guidelines on renal cell carcinoma [4, 5]. Residual disease and positive margins were defined histologically and governed the need for further treatment. Local recurrence was defined as new contrast enhancement within the RFA ablation zone or the RPN treated area on follow-up CT or MR on the basis of standard radiological criteria [5]. Tumour seeding within the renal bed (following partial nephrectomy) or along the needle tract (in patients treated with RFA) was analysed in a similar way to local recurrence. Metastasis was defined as evidence of biopsy-proven disease elsewhere in the body that has originated from the treated kidney. Disease-free survival was defined as the percentage of patients free from any recurrence and metastasis on follow-up [4, 5].

Baseline demographics were analysed using the Chi-square test for categorical variables and Fisher’s exact test as applicable. Continuous data that showed normal distribution, such as patient age, were evaluated using *t* test. Non-parametric data were analysed using the Mann–Whitney ‘U’ test. Disease-free Survival was analysed with the Kaplan–Meier method. Survival curves for the two groups were compared using the log-rank test and corresponding hazard ratios were calculated. Cox regression analysis (proportional hazards model) was used to produce a survival function that would predict the probability of the event of interest (e.g. local recurrence) occurring at a given time *t* for given values of the predictor variables (confounders). Regression covariates included baseline tumour size, PADUA score, age and type of treatment offered (RFA vs RPN). The StatsDirect (version 2.7) and the GraphPad Prism (version 6.0) software systems were used for statistical analysis, and a *p* value of <0.05 (at 95 % confidence interval) was taken as statistically significant.

## Results

Our sample consisted of 63 patients in each group, a total of 126 patients who met our inclusion criteria (Table 1). Table 2 demonstrates our sample’s baseline characteristics. The RPN group was significantly younger (*p* < 0.0001), and the majority of patients had an ASA score of ≤2 (*p* = 0.14). Mean tumour size was significantly different in the two groups (*p* = 0.0003). However, the PADUA score, which takes into account tumour size as well as anatomical characteristics that can affect complexity and complications of the treatment, showed no significant difference between the two groups (*p* = 0.69). Furthermore, the RFA group consisted of 16 cases with a single kidney as opposed to only one in the RPN group (*p* = 0.0002). The baseline renal function was significantly poorer in the RFA group (51.5 ± 20.0 mls/min/1.73 m^2^) compared to the RPN group (87.8 ± 15.1 mls/min/1.73 m^2^). In addition, the RFA group included two patients with documented von Hippel Lindau disease (both had retinal haemangioblastomas) and three cases with multiple small renal tumours (2, 2 and 4 discrete lesions, respectively, all <2.0 cm). All multifocal renal tumour cases had undergone unilateral total nephrectomy previously and were treated with sequential RFA sessions for nephron sparing purposes. The majority of tumours were histologically proven clear cell carcinomas in both RFA and RPN groups (Table 2).

**Table 2:** Baseline demographics

	RFA	RPN	*p* value
Group size, *n*	63	63	
Age (years), mean	61 ± 21	54 ± 7	<0.0001
ASA score/*n*; median (IQR)	2 (2–3)	2 (2–3)	0.14
1	1	3	
2	8	13	
3	6	6	
4	3	1	
Tumour size (cm), mean	2.11 ± 0.19 (range, 0.5–5.4)	2.88 ± 0.13 (range, 1.0–6.0)	0.0003
PADUA score, mean	7.27 ± 0.23	7.38 ± 0.16	0.69
Baseline eGFR	51.5 ± 20.0	87.8 ± 15.1	<0.0001
Single kidney, *n*	16/63	1/63	0.0002
Tumour histology (*n*)			
Clear cell	48	54	
Papillary	9	7	
Chromophobe	2	2	
Unspecified	4	0	

**Fig. 2:**
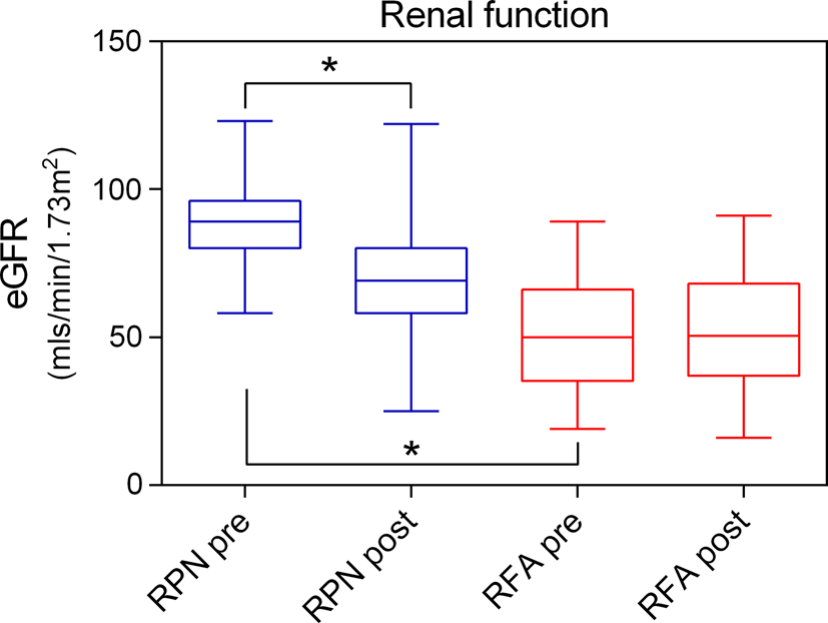
Boxplot of pre- and post-operative renal function (eGFR) in the RPN (*blue*) and RFA (*red*) groups. The baseline renal function was significantly poorer in the RFA group (*p < 0.05). Post-operative decline was significantly greater in patients treated with RPN (*p < 0.05)

Table 3 outlines the peri-procedural variables, and procedure-related complications calculated using the Clavien complications score. Mean drop in Hb concentration was significantly higher in the RPN group (*p* < 0.0001). Both Creatinine (Cr) increase and Glomerular Filtration rate (GFR) decrease were higher in the RPN group in keeping with a greater insult to the kidney. Early post-procedure renal function decline at 30 days was significantly less in RFA [(−0.8) ± 9.6 vs. (−16.1) ± 19.5 mls/min/1.73 m^2^; *p* < 0.0001]. Pre- and post-operative renal function in the 2 groups is illustrated in Fig. 2. Analysis of procedural variables revealed no conversions to open surgery and no blood transfusions in either group. Moreover, most RFA cases were performed as procedures with a single overnight stay as opposed to a median three-day hospital stay in the RPN group (*p* < 0.0001). Clavien complication grading system showed a similar minor complication rate with no significant difference between the two groups (Table 3).

**Table 3:** Peri-procedural outcomes

	RFA	RPN	*p* value
Hb change post procedure, (g/dL)	(−0.3) ± 1.5	(−1.8) ± 0.85	<0.0001
sCr change post procedure, (µmol/L)	(−6.3) ± 15.8	(−8.3) ± 18.8	0.36
eGFR change post procedure (mls/min/1.73 m^2^)	(−0.8) ± 9.6	(−16.1) ± 19.5	<0.0001
Length of hospital stay (days; median, IQR)	1 (1–1)	3 (2–3)	<0.0001
Minor complications (Clavien I and II)	4/63	10/63	0.15
Major complications (Clavien III and IV)	1/63	1/63	1.00
Clavien complication grade, (*n*)			
I	4	8	
II	0	2	
III	0	1	
IIIa	0	0	
IIIb	0	1	
IV	1	0	
IVa	0	0	
IVb	0	0	
V	0	0

The median follow-up for the RFA group was significantly longer than that for the RPN group, 4.0 vs. 1.5 year, respectively (*p* < 0.0001), because of the more recent commencement of the RPN service (Table 4). Mid- to long-term oncological outcomes including residual disease, local recurrence, metastasis, as well as need for new or repeat treatment were similar in the two groups and showed no statistically significant differences (Table 4). In two RFA cases, a residual enhancing tumour was identified on the immediate post-operative scan and was treated successfully with repeat ablation at 42 days and 2.5 months. In one RPN case, tissue biopsy showed positive margins, and further surgical excision was performed at the same time. Local recurrence was numerically higher in the RFA group, but not statistically significantly so (Table 4). In the RFA group, there were 4 recurrences (2 in single kidneys, one of which with multiple tumours at baseline) and 2 cases of tumour seeding that occurred after a median of 28 months (range, 8–84 months). There was no increased incidence of local seeding or recurrence in RFA-treated single kidneys (2 out of 16 vs. 4 out 47 cases, *p* = 0.64). Metastases developed in four patients (3 in RFA and 1 in RPN; *p* = 0.62) after a median period of 37 months (range, 9–59 months).

**Table 4:** Long-term clinical outcomes

	RFA	RPN	*p* value
Follow-up, months (median, range)	47.5 (11.8–80.2)	18.5 (6.2–29.5)	<0.0001
Residual disease/positive margins (*n*) (repeat treatment performed)	2/63	1/63	1.00
Local recurrence/tract seeding (*n*) (repeat ablation or nephrectomy)	6/63	1/63	0.11
Renal cancer metastasis (*n*)	3/63	1/63	0.62

**Fig. 3:**
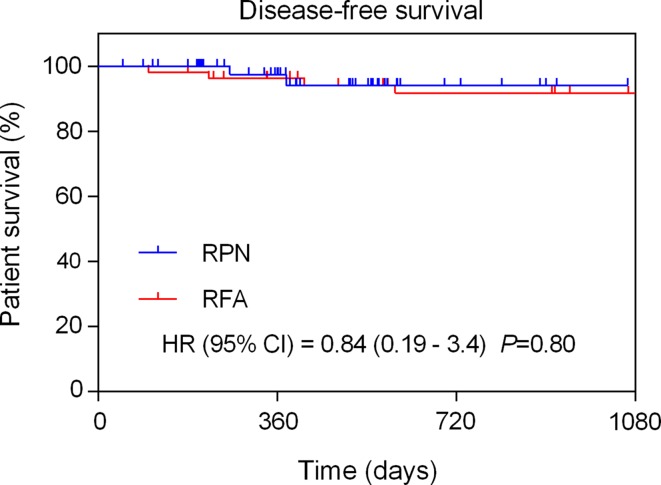
Comparative Kaplan–Meier analysis of disease-free survival between the RFA and RPN groups (unadjusted univariate hazard ratio noted)

Disease-free survival (DFS) was not significantly different between the two groups (HR = 0.84, 95 % Cl 0.19–3.4; *p* = 0.80; Fig. 3). Cox regression confirmed the results with a non-significant adjusted HR = 0.6 (95 % Cl 0.1–3.7; *p* = 0.60) in favour of RPN (adjustment for baseline tumour size, PADUA score, age and type of treatment offered). On the other hand, tumour size had a significant effect on DFS with increasing size found to be an independent predictor of local recurrence (Cox adjusted HR = 1.7; 95 % Cl 1.1–2.6 per cm of tumour size; *p* = 0.02). The adjusted hazard ratio (HR) derived from the Cox regression analysis is interpreted per unit of measurement, i.e. per cm of tumour size. Hence, assuming proportional hazards (Cox model), the hazard of recurrent disease would increase by 1.7 times per centimetre of original tumour size.

## Discussion

Open partial nephrectomy has long been considered the treatment of first choice for T1 small renal masses (SRMs) [18]. Recently, there has been a move towards less invasive procedures such as laparoscopic and robotic partial nephrectomy [19, 20]. RPN seems to be the currently more widely used method, in experienced centres, due to the added benefits offered by the da Vinci robot (such as improved precision and decreased range of motion) leading to decreased warm ischaemic times [21]. RFA is considered as an alternative treatment for T1 SRMs in those patients with significant comorbidities (ASA score >2) and advanced age [2, 5, 7]. In some cases, RFA is offered alongside RPN as an adjunct, to the surgical procedure according to baseline comorbidities. Moreover, the European Association of Urology (EAU) and the American Urology Association (AUA) guidelines recommend partial nephrectomy due to favourable long-term oncological outcomes and lack of long-term RFA outcomes [4–6]. However, recent RFA studies have shown promising oncological outcomes for carefully selected individuals, such as patients with significant comorbidities as well as patients who could have equally been candidates for RPN [1]. Due to the relative lack of studies where RFA is investigated as an alternative or adjunct to RPN in carefully matched patient groups, we felt the need to investigate this in detail [22].

By retrospectively comparing patients at a single tertiary centre, we were able to match tumour sizes, PADUA scores and comorbidity scores, and evaluate the mid- to long-term outcomes with either treatment option. Baseline demographics were well matched in both groups, but comorbid conditions were slightly more pronounced in the RFA group. Tumour size was significantly higher in the RPN group, while significantly more single kidneys were treated in the RFA group (Table 3). Overall disease-free survival (DFS) was similar in the two groups (HR = 0.91), with no statistically significant significance. One third of local recurrences observed in the RFA group (2 out of 6 cases) were due to tract seeding during ablation (confirmed radiologically), a well-known complication of the procedure (Table 3). As one of the goals of RFA is to spare normal renal parenchyma, and interventional radiologists do not seek to ablate with large margins, it is possible that this increases the risk of missed microscopic disease, whereas in RPN larger tissue margins are usually excised. The policy of minimizing the loss of normal renal parenchyma partly derives from the fact that RFA, unlike RPN, can be repeated with minimal discomfort and risk to the patient. The policy of sparing normal renal parenchyma is even more important in patients with single kidneys as one of the main aims of ablation is to minimize the risk of the patient requiring hemodialysis.

Follow-up was significantly longer in the RFA group because of a more recent establishment of the RPN service (*p* < 0.0001). It is notable that two local recurrences and two late metastases were identified in the RFA group after 3 years, which was beyond the maximum follow-up period in the RPN group. This may be due to the slow growth rate of the disease and clearly indicates the need for ongoing long-term follow-up (Table 4).

In addition, more minor complications were recorded in the RPN group, but local recurrence and/or seeding was more frequent in the RFA group (both non-significant trends). The baselines demographic results partly reveal the current treatment preferences among clinicians and patients (Table 2). For example, the median age and ASA comorbidities score show that RFA was offered to older patients and patients with more severe comorbidities (grades III–V). Furthermore, RFA was routinely offered to patients with von Hippel Lindau disease, multifocal tumours and single kidneys (mostly following contralateral nephrectomy), who may be associated with a higher risk of recurrent or new tumours. Notwithstanding the fact that RFA was offered to more frail patients with poorer baseline renal function, the mid- to long-term clinical outcomes compare well with those of robotic partial nephrectomy. On the basis of these results, a case can be made for offering this treatment to younger patients, those with fewer comorbidities and those who prefer a less invasive procedure [22]. Our results in relation to peri-operative complications and mid- to long-term oncological outcomes are similar to those of other individual studies solely examining RFA or RPN [23, 24].

Certain strengths as well as limitations were unique to our study and are worth discussing further. Firstly, our data were retrospectively collected and analysed, raising the possibility of selection bias. Arguably, the validity of our results may have been limited by the discrepancy in follow-up length between the two groups. This was outside this study’s control, due to the later establishment of RPN in comparison to RFA. The first RFA case performed at our centre was in 2004 as opposed to 2010 for the first RPN case. However, this was partly overcome by the more regular basis at which RPN is currently performed, giving us, thus, an adequate sample size. Secondly, our tertiary centre has certain referral patterns, for example, with referring urologists sending patients specifically for RPN or local urologists referring older and frailer patients for RFA. This might not be the case in other institutions, limiting the generalizability of our results. Thirdly, there was a significant number of lost to follow-up, especially in RFA cases. There are several factors contributing to this, most of which were outside of our control (such as patients followed up locally at other centres). This could have undermined our previously stated results or limited our sample of cases. Moreover, despite an adequate sample size, the low number of post-operative events limited the power of our statistical analysis.

A final limitation was the heterogeneous patient demographics of our two treatment groups. However, regression analysis was employed to adjust for the confounders. In addition, despite the promising oncological outcomes of both techniques and overall disease-free survival, it is worth noting that the success of both procedures is dependent on very sub-specialized training, not currently available at all tertiary training centres and requires expertise for selecting the right patient population that could potentially benefit from either procedure.

On the other hand, PADUA nephrometry scores enable comparison of our results with future studies. In addition, the use of a single technique for both RPN and RFA cases, performed by the same clinical team eliminated any variability in treatment success evaluation (e.g. operator or technique dependence). Furthermore, the fact that both Urology and Interventional Radiology departments were involved in this research as well as independent healthcare professionals (not affiliated with either department) minimized bias and strengthens the reliability of our results. Despite the above limitations, our study succeeded in confirming excellent oncological outcomes in RPN cases and emphasizing similar comparable outcomes with RFA in a selected group of patients. These results come with the added advantage that a minimally invasive approach offers: shorter hospital days, lower risk of intra-operative bleeding and potential better preservation of renal function; still little is known about long-term impact in residual renal function and adaptive kidney mechanisms with either technique. Therefore, RFA serves as an additional treatment option for the experienced urologist who can carefully recommend it to appropriately selected patients with small (T1) renal masses or as a future potential adjuvant treatment alongside RPN [25]. In line with our findings, comparable results between RFA and partial nephrectomy were recently reported from other centres both for localized stage 1a and for stage 1b renal tumours [6, 26].

In conclusion, both robotic-assisted partial nephrectomy and percutaneous radiofrequency ablation offer excellent oncological outcomes for the treatment of T1 RCC with low associated peri-operative morbidity. RFA was associated with fewer peri-operative complications and better preservation of renal function, whereas RPN had an insignificantly lower local recurrence rate. RFA could be offered alongside RPN for selected cases. Prospective randomized studies and subsequent meta-analyses in different age groups, PADUA classifications and histological variants will help confirm the interchangeable use of these two methods of treatment of small renal tumours.

## Abbreviations

ASA: American Society of Anaesthesiologists
AUA: American Urology Association
AMLs: Angiomyolipomas
Cr: Creatinine
DFS: Disease-free survival
EAU: European Association of Urology
GFR: Glomerular filtration rate
NCI: National Cancer institute
NSS: Nephron sparing surgeries
PADUA: Pre-operative aspects and dimensions used for an anatomical classification score
RFA: Radiofrequency ablation
RPN: Robotic-assisted partial nephrectomy
RCC: Renal cell carcinoma
SRMs: Small renal masses
WIT: Warm ischaemia time
